# Parent experiences and information needs relating to procedural pain in children: a systematic review protocol

**DOI:** 10.1186/s13643-017-0499-2

**Published:** 2017-06-06

**Authors:** Allison Gates, Kassi Shave, Robin Featherstone, Kelli Buckreus, Samina Ali, Shannon Scott, Lisa Hartling

**Affiliations:** 1 Alberta Research Centre for Health Evidence (ARCHE), University of Alberta, 11405-87 Avenue, Edmonton, Alberta T6G 1C9 Canada; 2 Department of Pediatrics, University of Alberta, 11405-87 Avenue, Edmonton, Alberta T6G 1C9 Canada; 3 Faculty of Nursing, University of Alberta, 11405-87 Avenue, Edmonton, Alberta T6G 1C9 Canada; 4 Women and Children’s Health Research Institute, University of Alberta, 11405-87 Avenue, Edmonton, Alberta T6G 1C9 Canada

**Keywords:** Procedural pain, Emergency medicine, Pediatrics, Information needs, Parents

## Abstract

**Background:**

There exist many evidence-based interventions available to manage procedural pain in children and neonates, yet they are severely underutilized. Parents play an important role in the management of their child’s pain; however, many do not possess adequate knowledge of how to effectively do so. The purpose of the planned study is to systematically review and synthesize current knowledge of the experiences and information needs of parents with regard to the management of their child’s pain and distress related to medical procedures in the emergency department.

**Methods:**

We will conduct a systematic review using rigorous methods and reporting based on the PRISMA statement. We will conduct a comprehensive search of literature published between 2000 and 2016 reporting on parents’ experiences and information needs with regard to helping their child manage procedural pain and distress. Ovid MEDLINE, Ovid PsycINFO, CINAHL, and PubMed will be searched. We will also search reference lists of key studies and gray literature sources. Two reviewers will screen the articles following inclusion criteria defined a priori. One reviewer will then extract the data from each article following a data extraction form developed by the study team. The second reviewer will check the data extraction for accuracy and completeness. Any disagreements with regard to study inclusion or data extraction will be resolved via discussion. Data from qualitative studies will be summarized thematically, while those from quantitative studies will be summarized narratively. The second reviewer will confirm the overarching themes resulting from the qualitative and quantitative data syntheses. The Critical Appraisal Skills Programme Qualitative Research Checklist and the Quality Assessment Tool for Quantitative Studies will be used to assess the quality of the evidence from each included study.

**Discussion:**

To our knowledge, no published review exists that comprehensively reports on the experiences and information needs of parents related to the management of their child’s procedural pain and distress. A systematic review of parents’ experiences and information needs will help to inform strategies to empower them with the knowledge necessary to ensure their child’s comfort during a painful procedure.

**Systematic review registration:**

PROSPERO CRD42016043698

**Electronic supplementary material:**

The online version of this article (doi:10.1186/s13643-017-0499-2) contains supplementary material, which is available to authorized users.

## Background

The World Health Organization asserts that each child has the right to health and the right to be spared avoidable pain [1]. In this regard, a substantial number of children and neonates have been underserved. Procedures frequently carried out in the emergency department (ED) represent one of the most common sources of acutely painful stimuli for children. These may include venipuncture, intravenous (IV) insertion and removal, capillary sampling, stapling and suturing, fracture reductions, oral and nasal suctioning, and tape removal [2]. Simply visiting the ED and the thought of a potentially painful procedure can provoke distress for children. In many cases, the pain and distress induced by these procedures is preventable or could be substantially relieved [3]. Though many evidence-based interventions are widely available to manage procedural pain in children and neonates, they remain severely underutilized in pediatric populations [4–7].

Inadequate management of procedural pain in children and neonates has been linked with numerous psychological and physiological consequences. In neonates, poorly managed procedural pain may have a lasting negative impact on their pain responses [8]. Infants who have experienced multiple painful procedures (e.g., heel lance) are more pain sensitive as adolescents and are more likely to report somatic symptoms than infants who did not experience multiple painful procedures [9, 10]. For children, poorly managed procedural pain may result in greater anxiety, pain, and avoidance of health care as adults [9]. Inadequate procedural pain management early in life may also decrease the effectiveness of adequate analgesia in subsequent procedures [11].

Watching their child undergo a painful procedure can also negatively impact the physiological and psychological wellbeing of parents [12]. This experience may lead to increases in heart rate, blood pressure, and self-reported anxiety [12]. Parent distress around their child’s procedure also positively predicts increased child pain and distress, suggesting that parents play an important role in their child’s procedural pain management [12]. Primary components of providing family-centered pediatric emergency care involve alleviating patient and family distress and fear around treatments, incorporating the family’s knowledge and preferences into their child’s treatment plan, and communicating information and providing the family with necessary education for their active involvement in their child’s care [13].

In the context of an unscheduled visit to the ED, children and their parents are particularly vulnerable; they may have no experience managing painful procedures and have little time to prepare for what is about to happen. Although also painful, community-based procedures like vaccinations are predictable and can be prepared for. Moreover, children who are inpatients or who are living with chronic conditions experience painful medical procedures much more frequently than otherwise healthy children in the ED. As such, their parents likely have very different experiences, knowledge, and information needs.

To date, research has focused primarily on child and/or caregiver outcomes pertaining to interventions for procedural pain management (e.g., distraction, topical anesthetics, breastfeeding). Recognizing the vulnerability of a parent whose child requires a painful medical procedure in the ED, we aim to design and implement relevant strategies to help parents understand and effectively manage their child’s pain and distress specific to this context. First, a comprehensive understanding of their experiences and information needs is required. To the authors’ knowledge, a comprehensive review of relevant literature has yet to be published on this topic. In response to this knowledge gap, the primary objective of the proposed study is to systematically review primary research, published since the year 2000, investigating parent information needs and experiences around children’s distress and pain during painful medical procedures in the ED. We aim to answer the following key research questions:

1. What are parents’ experiences surrounding the management of their child’s pain and distress during painful medical procedures in the ED?
2. What information do parents need to understand and manage their child’s pain and distress during a painful medical procedure in the ED?

## Methods

The proposed systematic review will be undertaken between July and November 2016, following an a priori protocol, as described herein. The protocol for the review was designed in accordance with the Preferred Reporting Items for Systematic Review and Meta-Analysis Protocols (PRISMA-P) guidelines (see Additional file 1) [14]. Any methodological changes from the protocol to the final systematic review will be documented in the subsequent manuscript and submitted for publication.

### Experiences and information needs

For the purposes of the proposed systematic review, we will operationalize experiences and information needs as follows. Experiences encompass how a parent feels (e.g., scared or calm; confident or nervous) and acts (e.g., supportive or withdrawn) during and immediately before and after their child undergoes a painful medical procedure and may include physical, emotional, and psychological sensations. Experiences are subjective and deeply personal, such that parents going through similar situations may react differently based on myriad factors (e.g., preparedness, knowledge, co-morbid stressors). Information needs include the content (i.e., topic), mode of delivery (e.g., electronic, paper-based, verbal), and amount of information that parents desire to receive about managing their child’s procedural pain. Information needs are not entirely distinct from experiences. How timely the information is and the context in which it is provided (e.g., noisy, calm) can impact how it is received. This, in turn, may impact a parent’s experience of the procedure and their ability to comfort their child.

### Search strategy

The search strategy will combine subject headings and keywords for concepts: pain, ED procedures and visits, parents, and information needs. The author team includes pain research (SA), systematic review methodology (LH), and knowledge translation (SS) specialists, as well as a medical research librarian (RF). The team convened to decide on relevant keywords and subject heading to include in the search, including procedures that are common to the ED setting (e.g., venipuncture, IV insertion). Database searches will be limited to English language records published since January 2000. The author team selected 2000 as the start date for the search after conducting a preliminary investigation of systematic reviews on pediatric procedural pain in the hospital setting. To our knowledge, the earliest systematic review was conducted in 1999 [15] and reported small sample sizes and inconsistent findings from included studies. Since then, the evidence-base for pediatric pain management has improved significantly [16, 17]. In order to identify parents’ experiences and information needs as they pertain to current practices in pediatric pain care, we will exclude studies published prior to 2000, as these are likely not reflective of the current parental experience.

We will search the following online databases: Ovid MEDLINE In-Process & Other Non-Indexed Citations and Ovid MEDLINE (1946 to present), Ovid PsycINFO (1987 to present), CINAHL via EBSCOhost (1937 to present), and PubMed via NCBI Entrez (electronic publications only). These databases will provide coverage of literature in the health and biomedical sciences, as well as behavioral sciences, psychology, and mental health. To identify studies not published in indexed journals, we will search selected gray literature sources including ProQuest Dissertations and Theses Global, and conference proceedings (e.g., Canadian Pain Society, American Pain Society, International Association for the Study of Pain, International Symposium on Pediatric Pain). Authors of conference abstracts will be contacted via email to inquire as to the availability of related full-text records. To further reduce the likelihood that a relevant article is omitted, we will review the reference lists of included articles. Our search strategy for Ovid MEDLINE is shown in an additional file (Additional file 2).

### Study selection

Search results will be uploaded to EndNote (*v*. X7, Thomson Reuters, Toronto, Canada), a reference management tool accessible locally to the study team members. All duplicates will be removed from the EndNote Library. Two independent reviewers will screen records for inclusion. In the first-level screening, the reviewers will screen the records by title and abstract for relevance. Eligible records will be retrieved and will undergo second-level screening where full-text studies will be reviewed for inclusion. Disagreements between reviewers will be resolved through discussion or involvement of a third-party with subject area expertise. All decisions regarding the exclusion of studies will be documented and reported on. Inclusion and exclusion criteria for article selection are detailed in Table 1. The study team will convene monthly throughout the study selection process to discuss findings to date and refine the inclusion and exclusion criteria, if necessary. A flow diagram, adapted from the PRISMA guidelines [18], will be developed to demonstrate the movement of articles throughout the review process.

**Table 1:** Inclusion and exclusion criteria for the selection of studies relevant to the review

Characteristic	Inclusion criteria	Exclusion criteria
Study design	Primary research Qualitative or quantitative study designs	Reviews and overviews^a^ Opinion pieces (i.e., comments, editorials, letters)
Publication date	Articles published from January 2000 to present	Articles published prior to January 2000
Language	Articles published in English	Articles published in any language other than English
Population	Parents of infants, children or youth aged 0 to 18 years Human populations	Populations other than parents of infants, children or youth aged 0 to 18 years Animal studies
Procedures	Mildly (e.g., oral and nasal suctioning), moderately (e.g., dressing or tape removal, wound irrigation) and severely (e.g., venipuncture, phlebotomy, intravenous insertion, lumbar puncture, suprapubic aspiration) painful procedures [2] common to the emergency medicine setting	Procedures less common to the emergency medicine setting (e.g., vaccinations administered in the community, in-patient procedures); urinary catheter insertions; endoscopies; tracheotomies; procedural sedation
Outcomes	Articles describing parent experiences immediately before and/or after, and/or during the painful procedure, and their information needs with regard to managing their child’s distress and pain	Articles describing and/or evaluating tools or education programs that parents can use to manage their child’s distress and procedural pain

^a^If the topic of a review or overview is relevant, we may make use of these sources by checking the reference lists to identify primary studies for inclusion

### Data extraction

Data extracted from each article will include publication year, country, population and sample size, and study design. We will also extract data on procedure type, clinical setting in which the procedure was conducted, and child age to determine the clinical context of included studies. In accordance with the primary objective of the proposed review, we will extract the main findings as they pertain to parent experiences and information needs related to their child’s procedural pain and how these were measured in each respective study. The data extraction for each study will be completed by one reviewer using a data extraction form developed by the research team. A second reviewer will check the data extraction for accuracy and completeness. Any disagreements between the two reviewers will be resolved via discussion to reach consensus. The data will be presented using a summary table that describes the included studies.

### Data synthesis

Based on our preliminary searches, we expect that the majority of the studies included within this review will be qualitative. Studies reporting quantitative findings may also be included. For qualitative studies, presenting the findings in a tabular format will not adequately represent the richness of the data. Therefore, we plan to employ a thematic synthesis approach [19] to generate key analytical themes describing the amalgamated findings from the included articles. Thematic synthesis is the recommended approach for summarizing qualitative data from systematic, comprehensive reviews of the literature [20]. All data from the “Results” or “Findings” sections of each qualitative study will first be extracted verbatim to NVivo (*v*. 10, QSR International, Melbourne, Australia) data management software. After reading through the text, we will employ line-by-line coding, assigning one or more codes to each line of text. These codes will then be organized into analytical themes. The coding will be inductive, as to not impose any a priori theories or expectations onto the data, allowing the themes to emerge from the data themselves. To reduce the risk of bias (e.g., confirmative bias), the thematic synthesis will be conducted by one reviewer, while a second reviewer will appraise the preliminary coding. A debriefing meeting will then be used to ensure transparency in the coding and agreement between the reviewers. The two reviewers will ultimately agree on the final overarching themes that emerge from the analyses, which will be presented narratively. The findings of the quantitative studies will be presented either in a tabular format or narratively, depending on the quantity of records identified. For example, the results of surveys or validated questionnaires will be described with proportions, means and standard deviations, or any other measures that are reported in the original publication. Because we will be excluding intervention studies, we do not plan to conglomerate the findings of the studies using a meta-analytic approach.

### Quality of evidence and risk of bias

Risk of bias and methodological quality will be determined for each study utilizing tools specific to qualitative and quantitative methodologies. Two reviewers will independently assess each study using the appropriate assessment tool based on study methodology. Discrepancies in decisions will be resolved through discussion.

Risk of bias and methodological quality of individual qualitative studies included in our review will be assessed using CASP, the Critical Appraisal Skills Programme Qualitative Research Checklist [21]. This commonly used appraisal tool broadly seeks to assess the validity and local utility of the results of qualitative research [20]. Ten components of a study are systematically assessed, including the aims of the research, methodology, research design, recruitment strategy, data collection, relationship between researcher and participant, ethical issues, data analysis, findings, and overall value of the research. All items will be reported on.

Risk of bias and methodological quality of individual quantitative studies included in our review will be assessed using the Quality Assessment Tool for Quantitative Studies [22]. This rating tool assesses studies on eight domains: selection bias, study design, confounders, blinding, data collection methods, withdrawals and drop-outs, intervention integrity, and analyses. Based on assessments of each domain, the article is then ranked globally as strong, moderate, or weak. All items and global rankings will be reported on.

### Confidence in cumulative evidence

Regardless of the results of the risk or bias and quality assessments, all relevant studies will be included in the review. We will report the conglomerate findings of included studies in light of the results of these assessments. For qualitative studies, those that score “yes” on items 1–3 (e.g., clear statement of the aims of the research, qualitative methodology is appropriate, and research design is appropriate) will be considered to be of adequate quality. The findings of lower quality studies will thus have less bearing on the main emergent themes from the qualitative analysis. For the quantitative studies, those ranked “strong” will, accordingly, have greater influence on the cumulative evidence reported as compared to those ranked “moderate” or “weak.”

## Discussion

Pediatric procedural pain has been widely reported as undermanaged throughout the published literature [3, 5, 7]. Caregivers of children undergoing painful procedures in the ED demonstrate interest and commitment to ensuring their child has a pain- and distress-free experience. The vast majority of caregivers express that, if possible, they would choose to make their child’s procedure painless and would be willing to spend additional time in the ED if required to make this possible [23]. Emergency departments across North America continue to face time and resource constraints, including long wait times and high patient volumes [24–27]. Caregivers represent a captive audience who is motivated to eliminate the distress and pain their children face when undergoing painful procedures and, in most cases, has the time to have a role in doing so.

Our proposed review will establish a more comprehensive understanding of parent experiences and information needs relating to children’s procedural pain. We will apply the findings to the development and evaluation of tailored, innovative knowledge translation tools to facilitate parents taking on an engaged role in managing their child’s distress and pain related to medical procedures in the ED. Besides parents, we plan to disseminate the findings of this review to other relevant stakeholders, who may include health care practitioners, academics with an interest in pediatric procedural pain management, and experts in public health, health promotion, and knowledge translation. A multimodal dissemination plan (e.g., publication, presentations, knowledge translation tools) will ensure a broad reach.

## Additional files

Additional file 1: Completed PRISMA-P Checklist. (DOCX 28 kb)

Additional file 2: Sample Search Strategy for Ovid MEDLINE. (DOCX 16 kb)

## Abbreviations

IV: Intravenous
CASP: Critical Appraisal Skills Programme
ED: Emergency department
PRISMA: Preferred Reporting Items for Systematic Reviews and Meta-Analyses
PRISMA-P: Preferred Reporting Items for Systematic Review and Meta-Analysis Protocols
