# Supplementary material for: Parent experiences and information needs relating to procedural pain in children: a systematic review protocol
Source: Syst Rev. 2017 Jun 6;6:109. doi: 10.1186/s13643-017-0499-2 (PMC5461670; doi:10.1186/s13643-017-0499-2)
Supplement: Supplementary file 2 — Sample Search Strategy for Ovid MEDLINE. (DOCX 16 kb) [file 13643_2017_499_MOESM2_ESM.docx]

Sample Search Strategy for Ovid Medline

Database: Epub Ahead of Print, In-Process & Other Non-Indexed Citations, Ovid MEDLINE(R) Daily and Ovid MEDLINE(R) 1946 to Present

Strategy:

| 1. Anxiety/  2. Fear/  3. exp Pain/  4. Pain Perception/  5. Panic/  6. Stress, Psychological/  7. (afraid* or anxi * or fear* or distress* or pain* or panic* or scare* or scary or stress* or terrif*).tw,kf.  8. or/1-7 [Combined MeSH & text words for pain]  9. exp Administration, Intravenous/  10. Blood Specimen Collection/  11. Casts, Surgical/  12. exp Emergency Service, Hospital/  13. Fecal Impaction/  14. Gynecological Examination/  15. Injections/  16. Injections, Intramuscular/  17. Injections, Intravenous/  18. exp Injections, Spinal/  19. Needles/  20. Phlebotomy/  21. Punctures/  22. Spinal Punctures/  23. Suction/  24. Sutures/  25. ((access* or implant* or insert* or remov*) adj3 (intravenous* or IV or line* or port* or tape or tube*)).tw,kf.  26. ((acute care or critical care or emergency or trauma or urgen*) adj2 (cent* or department* or room* or unit* or ward*)).mp.  27. (blood adj2 (draw* or sampl* or test*)).tw,kf.  28. (blood work or bloodwork).tw,kf.  29. ((bone* or fracture* or limb*) adj5 (cast* or reduc* or set*)).tw,kf.  30. capillary sampl*.tw,kf.  31. (exam* adj3 (gyn* or pelvic or vagina*)).tw,kf.  32. ((extract* or remov*) adj2 (foreign bod* or foreign object*)).tw,kf.  33. ((extract* or peel* or remov*) and (cardiac lead* or chest lead*)).tw,kf.  34. ((faeces or fecal or feces or manual or rectal or rectum) adj2 (dis-impaction or disimpaction or impaction)).tw,kf.  35. injection*.tw,kf.  36. ((intravenous or IV) adj line?).tw,kf.  37. (laceration* adj2 repair*).tw,kf.  38. ((lumbar or spinal) adj2 (puncture* or tap*)).tw,kf.  39. needl*.tw,kf.  40. ((oral or nasal) adj3 suction*).tw,kf.  41. (pain* adj2 procedur*).tw,kf.  42. ((pain* or sore or tender) adj3 (ultra-so* or ultraso*)).tw,kf.  43. phlebotom*.tw,kf.  44. procedur*.ti.  45. procedur*.ab. /freq=2  46. sutur*.tw,kf.  47. stapl*.tw,kf.  48. stitch*.tw,kf.  49. (veni puncture* or venipuncture*).tw,kf.  50. (wound* adj2 irrigat*).tw,kf.  51. or/9-50 [Combined MeSH & text words for common ER procedures]  52. and/8,51 [Combined concepts for painful procedures]  53. Caregivers/  54. Family/  55. Parent-Child Relations/  56. Parenting/  57. exp Parents/  58. (care giver* or caregiver* or carer* or guardian*).tw,kf.  59. (families* or family* or father* or mother* or parent*).tw,kf.  60. or/53-59 [Combined MeSH & text words for caregivers]  61. Attitude to Health/  62. Caregivers/ed, px  63. exp Consumer Health Information/  64. Decision Making/  65. Evidence-Based Practice/  66. Focus Groups/  67. Health Education/  68. Health Knowledge, Attitudes, Practice/  69. exp Information Literacy/  70. Information Seeking Behavior/  71. Information Services/  72. Parents/ed, px  73. exp "Patient Acceptance of Health Care"/  74. Patient Compliance/  75. "Patient Education as Topic"/  76. Patient Participation/  77. Patient Satisfaction/  78. Personal Satisfaction/  79. "Quality of Life"/  80. "Retention (Psychology)"/  81. "Surveys and Questionnaires"/  82. accept*.tw,kf.  83. (adhere* or nonadhere*).tw,kf.  84. attitude*.tw,kf.  85. belie*.tw,kf.  86. (complian* or comply or noncomplian*).tw,kf.  87. comprehen*.tw,kf.  88. concern*.tw,kf.  89. (co-operat* or cooperat*).tw,kf.  90. (educat* or instruct* or learn* or perform* or train* or teach* or taught*).tw,kf.  91. experience*.tw,kf.  92. focus group*.tw,kf.  93. health litera*.tw,kf.  94. inform*.ab. /freq=3  95. inform*.ti,kf.  96. interview*.tw,kf.  97. know*.ab. /freq=3  98. know*.ti,kf.  99. misconce*.tw,kf.  100. opinion*.tw,kf.  101. participat*.tw,kf.  102. perce*.tw,kf.  103. perspective*.tw,kf.  104. prefer*.tw,kf.  105. (recall* or remember* or retain* or retention*).tw,kf.  106. satisf*.tw,kf.  107. QoL.tw,kf.  108. quality of life.tw,kf.  109. questionnaire*.tw,kf.  110. uncertain*.tw,kf.  111. underst*.tw,kf.  112. view*.tw,kf.  113. or/61-112 [Combined MeSH & text words for information needs]  114. and/52,60,113 [Combined concepts for procedural pain, caregivers and information needs]  115. exp Adolescent/  116. exp Child/  117. Child Behavior/  118. Hospitals, Pediatric/  119. exp Infant/  120. exp Minors/  121. exp Pediatrics/  122. (adolescen* or boy* or child* or girl* or preschool* or school age* or schoolchild* or teen* or toddler*).mp.  123. (baby* or babies or infant* or infancy or neonat* or newborn* or postmatur* or prematur* or preterm*).mp.  124. (paediatric* or peadiatric* or pediatric*).mp.  125. or/115-124 [Combined MeSH & text words for children]  126. 114 and 125 [Child filter]  127. animals/ not (animals/ and humans/)  128. (animal* or bovine or canine* or cat or cats or dog or dogs or feline* or hamster* or mice or monkey* or mouse or pig or piglet* or pigs or porcine or primate* or rabbit* or rat or rats or rodent or rodents or sheep or swine or swines or zebrafish*).ti.  129. 126 not (127 or 128) [Human filter]  130. (comment or editorial or news or newspaper article).pt.  131. (letter not (letter and randomized controlled trial)).pt.  132. 129 not (130 or 131) [Opinion pieces excluded]  133. limit 132 to yr=2000-current  134. limit 133 to english  135. remove duplicates from 134 |
| --- |
